# Cord Blood Metabolome and BMI Trajectory from Birth to Adolescence: A Prospective Birth Cohort Study on Early Life Biomarkers of Persistent Obesity

**DOI:** 10.3390/metabo11110739

**Published:** 2021-10-28

**Authors:** Tingyi Cao, Jiaxuan Zhao, Xiumei Hong, Guoying Wang, Frank B. Hu, Xiaobin Wang, Liming Liang

**Affiliations:** 1 Department of Biostatistics, Harvard T.H. Chan School of Public Health, 677 Huntington Avenue, Building II 4th Floor, Boston, MA 02115, USA; tingyi_cao@hsph.harvard.edu (T.C.); jzhao1@hsph.harvard.edu (J.Z.); 2 Center on the Early Life Origins of Disease, Department of Population, Family and Reproductive Health, John Hopkins University Bloomberg School of Public Health, Baltimore, MD 21205, USA; xhong3@jhu.edu (X.H.); gwang24@jhu.edu (G.W.); 3 Department of Nutrition, Harvard T.H. Chan School of Public Health, 677 Huntington Avenue, Building II 3rd Floor, Boston, MA 02115, USA; frank.hu@channing.harvard.edu; 4 Channing Division of Network Medicine, Department of Medicine, Brigham and Women’s Hospital and Harvard Medical School, 181 Longwood Avenue, Boston, MA 02115, USA; 5 Department of Epidemiology, Harvard T.H. Chan School of Public Health, 677 Huntington Avenue, Building II 2nd Floor, Boston, MA 02115, USA; 6 Department of Pediatrics, Johns Hopkins University School of Medicine, Baltimore, MD 21205, USA

**Keywords:** metabolomics, cord blood, growth trajectory, childhood obesity, body mass index

## Abstract

There is increasing recognition on the role of early life metabolic programming in childhood obesity. This study sought to investigate whether newborn cord blood metabolome can predict future BMI. It included 946 children in the Boston Birth Cohort, a sample of high-risk yet understudied US urban, low-income, predominantly Black and Hispanic children, who were enrolled at birth and followed prospectively up to age 18 years. A total of 376 metabolites were measured in cord blood plasma. Longitudinal BMI trajectories were defined and categorized into three distinct patterns: early onset overweight and obesity (early-OWO), late onset OWO (late-OWO), and normal weight trajectory (NW). Multinomial logistic regression models were used to identify metabolites individually or as network modules associated with BMI trajectories. Of the 946 children, 388, 254, and 304 were classified as early-OWO, late-OWO, and NW, respectively. Of the seven co-metabolomic network modules defined, two were inversely correlated with early-OWO. Among the 68 metabolites within the two modules, 22 triacylglycerols and diacylglycerols were negatively associated with early-OWO; 5 cholesterol esters were positively associated with early-OWO. In this prospective birth cohort, we demonstrated distinctive longitudinal BMI trajectories and identified multiple cord plasma metabolites in relevant biological pathways that were associated with early-OWO.

## 1. Introduction

The high prevalence (17%) of childhood obesity and its strong tendency to persist into adulthood with increased comorbidity and mortality have rendered it a present and future major public health burden in the United States [1,2,3]. Obesity in older children and adults is difficult to treat, while various early school-based and home-based interventions have proven to be effective in preventing childhood obesity to some extent [4]. To improve our ability to prevent obesity, it is crucial to identify early life biomarkers to better assess a child’s future risk of obesity so as to intervene at the earliest possible stage.

Since the cord plasma metabolite profile may reflect the transfer of maternal metabolites (in utero exposure) and fetal metabolic state [5], there is growing interest about whether cord plasma metabolite signatures can serve as biomarkers of early-life risk for metabolic diseases such as obesity later in life [5,6]. Indeed, available studies support this possibility. For example, studies discovered distinctive cord metabolic profiling for newborns with low birthweight [7,8]. Lu et al. reported strong positive associations between cord plasma LPC 14:0, 16:1, and 18:1 and birthweight [9]; and Kadakia et al. found that cord plasma branched-chain amino acids and ketone body metabolites were positively correlated with newborn adiposity [5]. Studies have also examined associations of cord plasma metabolites (e.g., tryptophan, acetaminophen, phospholipids) with postnatal weight gain, childhood obesity risk early in life at age 3–5 years, and young age-at-onset of type 1 diabetes [10,11,12]. To date, previous studies mostly focused on associations of cord metabolomics with birth outcomes or with child postnatal metabolic outcomes at limited age groups.

The central focus of this study was to investigate the association of individual cord plasma metabolites and their combined patterns with the longitudinal body mass index (BMI) trajectories. This study sought to advance the field by overcoming several limitations of previous studies. For instance, there is a lack of studies on long-term associations between cord plasma metabolites and children’s BMI; and none of the previous studies examined longitudinal BMI trajectory from birth to adolescence, which is important for risk assessment, prediction, and prevention. If only focusing on the BMI at a given age (one single time point), like most previous studies did, one cannot differentiae the clinical course of BMI evolution, for example if the obesity was early onset or late onset. Such insight may shed light on possible etiology of obesity and inform screening and intervention strategies. In addition, most studies examined single metabolite-BMI association, and few studies systematically examined the combined effect of cord metabolites as network modules, which is important given we know the biological system and its elements are inter-connected. Furthermore, most previous studies had relatively small sample sizes and few were conducted in US high-risk but understudied populations such as Blacks.

## 2. Results

### 2.1. Longitudinal Trajectory Analysis: Categorizing Longitudinal BMI Trajectories

**Figure 1:**
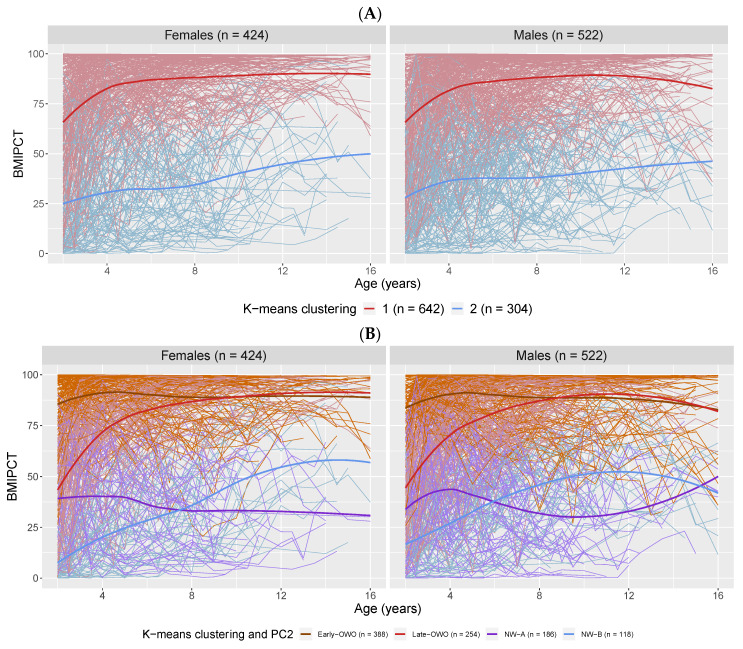
Longitudinal trajectory analysis: Plot of BMI trajectories for individual subjects (thin lines) colored by defined longitudinal BMI trajectories via k-means clustering into two groups (**A**) and via combining k-means clustering with PC2 information into 4 groups (**B**). Thick lines are the LOWESS smoothing curves for each group of subjects.

K-means clustering divided the 946 children into two clusters with 642 participants in cluster 1 and 304 participants in cluster 2. Supplementary Figure S1 shows the principal component analysis (PCA) plot of the two clusters of children and demonstrates that the k-means clustering was mainly based on the first principal component (PC1). The BMI percentiles (BMIPCT) trajectories in Figure 1A reveal that children in cluster 1 had overall higher BMI (65.7% clinically obese or overweight at last visit) than children in cluster 2 (0.98% clinically obese or overweight at last visit); thus, the k-means clustering result could be treated as a crude measure of children’s longitudinal BMI trajectories. While PC1 accounted for most of the variance (87.6%) in BMI trajectories, the second principal component (PC2) also explained 7.2% of the variance (Supplementary Figure S1). Therefore, we dichotomized PC2 around zero for children in cluster 1 and 2, respectively, to further divide participants into four subgroups. Figure 1B illustrates the BMIPCT trajectories of these four groups and shows that negative PC2 corresponded to a sharp increase in BMI at early ages, while positive PC2 implied relatively smooth longitudinal trajectories. Since k-means clustering together with PC2 could distinguish participants’ longitudinal BMI patterns in a more refined fashion, we considered this to be the outcome that best represented children’s BMI trajectories; as such, from this point we referred to these four groups as: early onset obese or overweight (OWO) for k-means cluster 1 + positive PC2 (n = 388), late onset OWO for k-means cluster 1 + negative PC2 (n = 254), normal weight trajectory A (NW-A) for k-means cluster 2 + positive PC2 (n = 186), and normal weight trajectory B (NW-B) for k-means cluster 2 + negative PC2 (n = 118).

Table 1 presents the characteristics of mother–infant dyads stratified by these four groups. Maternal characteristics were comparable among the four groups except for age at delivery (*p* = 0.038), race (*p* = 0.030), maternal OWO (*p* < 0.001), proportion with Cesarean section (*p* < 0.001), and breastfeeding (*p* = 0.029). Since the grouping was based on children’s BMI trajectories, the four groups of children differed in birthweight and growth outcomes at last visit (height, weight, BMI) as expected (*p* < 0.001).

**Figure 2:**
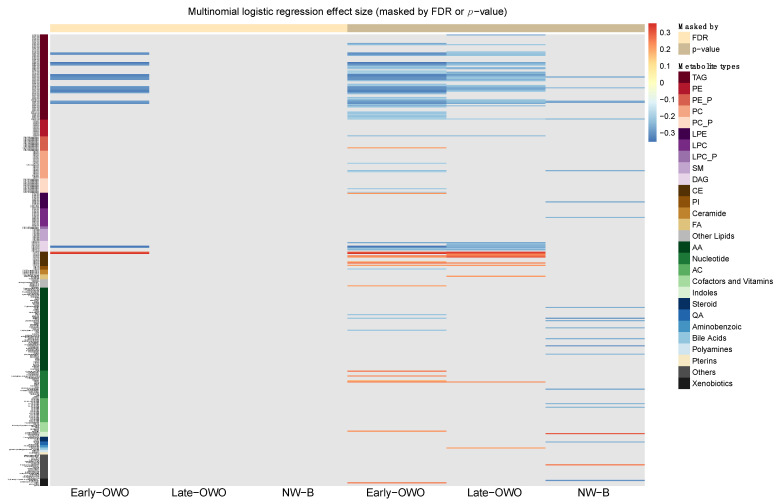
Longitudinal trajectory analysis: Heatmap of the effect size of metabolites on BMI trajectory (4 groups) masked by non-significance on the horizontal axis. The vertical axis represents the 376 metabolites which are ordered based on metabolite types. The effect size of the association between each metabolite and child BMI trajectory is the beta coefficient from the multinormal logistic regression (reference group is NW-A, i.e., the purple line in Figure 1B). The color scheme for effect size is continuous such that red and blue indicate positive and negative associations, respectively. The intensity of the color represents the magnitude of the association. For the first 3 columns, the grey color indicates where the overall effect of that metabolite at that BMI trajectory is not statistically significant after adjusting for multiple hypothesis testing across all 376 metabolites (FDR > 0.05); for the last 3 columns, the grey color indicates where the overall effect of that metabolite at that BMI trajectory is not statistically significant without adjusting for multiple hypothesis testing (*p* > 0.05).

Next, we fitted multinomial logistic regression models for each individual metabolite using this BMI trajectory-based grouping as the outcome. Figure 2 shows the effect size of each of the 376 metabolites on children’s BMI trajectories masked by either false discovery rate (FDR) or *p*-value (detailed model results listed in Supplementary Table S1). We made three comparisons. For the comparison between early-OWO and NW-A children (columns 1 and 4 in Figure 2), 76 metabolites had significant associations with a difference in BMI trajectories (*p* ≤ 0.05) and 23 remained significant after adjusting for multiple testing (FDR ≤ 0.05); for the comparison between late-OWO and NW-A children (columns 2 and 5 in Figure 2), 49 metabolites were marginally significant (*p* ≤ 0.05) though none passed multiple testing correction (FDR ≤ 0.05); for the comparison between NW-B and NW-A children (columns 3 and 6 in Figure 2), only 22 metabolites were marginally significant (*p* ≤ 0.05) and none passed multiple testing correction (FDR ≤ 0.05). The direction and size of the metabolites’ effects were consistent across the three comparisons, with TAGs and DAGs having negative associations with higher BMI, and CEs having positive correlations with higher BMI. The lack of significant associations between metabolites and children’s BMI trajectories for those in the NW-B group as compared to the reference NW-A group suggested we could collapse these two subgroups into one group, which mostly included children with normal (non-OWO) weight trajectories.

The modified categorization of the 946 children was meaningful from a clinical perspective: the first group included children with early onset OWO (k-means cluster 1 + positive PC2, n = 388), denoted as “early-OWO”; the second group included children with late onset OWO (k-means cluster 1 + negative PC2, n = 254), denoted as “late-OWO”; and the third group included children with normal (non-OWO) weight trajectories (k-means cluster 2, n = 304), denoted as “NW”. The following analyses were focused on comparing these three trajectory groups.

### 2.2. Longitudinal Trajectory Analysis: Metabolite Modules and BMI Trajectory Association

To identify cord metabolomic networks, the 376 metabolites were grouped into seven modules using the WGCNA (weighted correlation network analysis) package [13] based on correlation between metabolite pairs and assigned a color, while 58 metabolites not grouped into any module were labeled as “grey” (Supplementary Table S2). Multinomial logistic regression models were fitted for the three trajectory groups (early-OWO and late-OWO vs. NW) on the PC1 of each metabolite module. Table 2 shows the adjusted odds ratios with *p*-values and FDRs for each metabolite module. For the comparison between early-OWO and NW, the red (n = 25: 20 TAGs, 3 CEs, 1 DAG, and 1 PC) and brown (n = 43: 28 TAGs, 7 DAGs, 7 CEs, and 1 PE) metabolite modules were identified as significant after adjusting for multiple testing (FDR ≤ 0.05); for the comparison between late-OWO and NW, the same two metabolite modules showed up to be marginally significant (*p* ≤ 0.05) but did not pass multiple testing correction (FDR ≤ 0.05).

**Figure 3:**
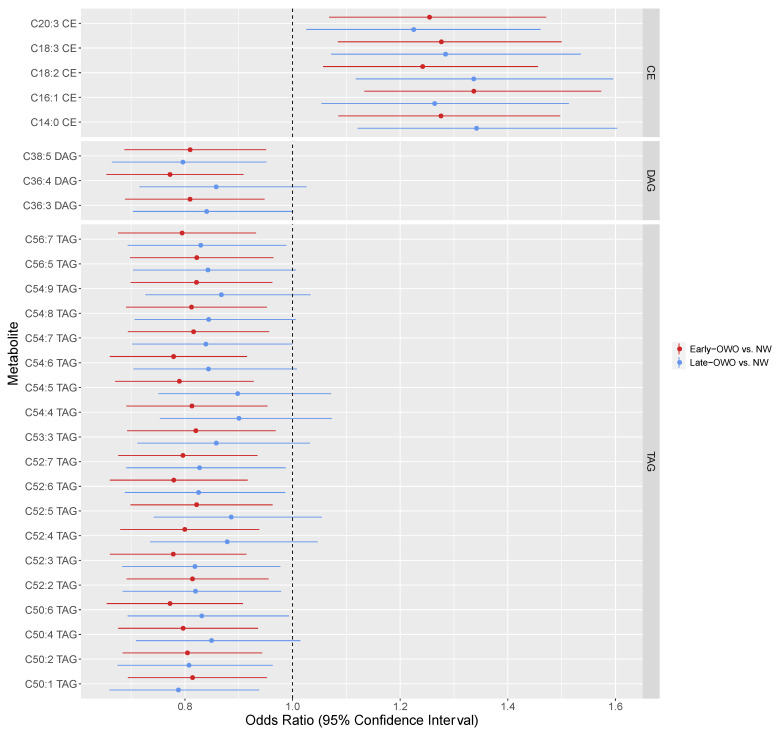
Longitudinal trajectory analysis: Forest plot showing associations of individual cord metabolites within each significant cluster (as identified in Table 2 with *p*-value ≤ 0.05 for either comparison) with child BMI trajectory groups (early-OWO and late-OWO as compared to NW) from multinomial logistic regression model results. Only metabolites with FDR of early-OWO vs. NW comparison less than or equal to 0.05 are listed here (results for all 68 metabolites in the 2 candidate metabolite modules identified in Table 2 are listed in Supplementary Table S3). Within each panel (metabolite type), individual metabolites are ordered based on FDR of early-OWO vs. NW comparison. FDR accounts for multiple hypothesis testing across all 68 metabolites in the 2 candidate metabolite clusters identified in Table 2.

We further analyzed the 68 individual metabolites in these two modules (red and brown) by testing their individual associations with the three BMI trajectory groups. Multinomial logistic regression models were fitted for the three trajectory groups (early-OWO and late-OWO vs. NW) on each of the 68 metabolites, respectively. Figure 3 is a forest plot showing the adjusted odds ratios with 95% confidence intervals for the top metabolites (FDR ≤ 0.05 for either of the two comparisons), while Supplementary Table S3 lists the model results for all 68 candidate metabolites. After accounting for multiple testing, only 2 metabolites were significant for the comparison between late-OWO and NW, but 27 metabolites showed up to be significant when comparing early-OWO with NW. As illustrated in the three panels of Figure 3, these 27 metabolites were all either TAGs, DAGs, or CEs. Among them, TAGs and DAGs were negatively associated with BMI (odds ratio < 1) while CEs were correlated with higher BMI (odds ratio > 1). Results for each of the 376 metabolites are provided in Supplementary Table S4.

### 2.3. Longitudinal Trajectory Analysis: Sensitivity Analysis

We carried out sensitivity analysis to explore whether metabolites’ effects differed by sex. Supplementary Figure S2 shows the effect size of each of the 376 metabolites for females and males, respectively. The heatmap was not masked by the *p*-value or FDR of the metabolite-by-sex interaction term because fewer than 10 metabolites had a significant interaction term (*p* ≤ 0.05) for any of the three comparisons in the multinomial logistic regression model, and they were no longer significant after correction for multiple testing. Supplementary Table S5 shows the association results between each of the seven metabolite modules and the BMI trajectories (early-OWO and late-OWO compared with NW) along with the interaction term *p*-value and FDR. No metabolite modules appeared to have significant effects after multiple testing correction. However, two modules were marginally significant (likelihood ratio test LRT *p* ≤ 0.05) and they were the brown and red modules which were consistent with the main analyses. The 68 metabolites within the two modules were further analyzed individually. Supplementary Table S6 shows the multinomial logistic regression model results for each of the 68 metabolites along with their interaction term *p*-values and FDR. Only one metabolite had a marginally significant interaction term (*p* ≤ 0.05) for each of the two comparisons, but none remained significant after adjusting for multiple testing. Therefore, the sensitivity analysis demonstrated that neither the effects of single metabolites nor the effects of metabolite modules differed significantly between sex.

### 2.4. Time-Window Specific Analysis: Individual Metabolites

**Figure 4:**
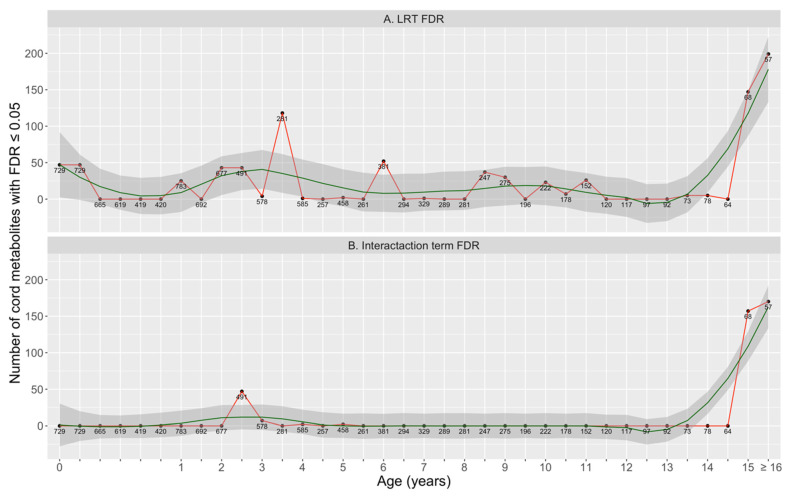
Time-window specific analysis: Number of significant metabolites at each time-window based on LRT FDR (**A**), number of metabolites that have significantly different effects between two sexes at each time-window based on metabolite-by-sex interaction term FDR (**B**). Annotated numbers are the sample size (number of subjects) available at each time-window. Green lines are the LOWESS smoothing curves. Potential critical growth ages (peaks in panel A) are around ages 0 (birth), 1–3.5, 6, 8–11, and ≥15.

We divided children’s repeated measurements of BMI from birth to age 18 into 36 disjoint time-windows (details in Methods Section 4.3.1 below). For each of the 36 time-windows, respectively, we fit multivariate linear regressions to test the associations between every cord metabolite and children’s BMI at that time-window. Figure 4A shows the number of metabolites that were significantly associated with BMI along time (LRT FDR ≤ 0.05). The peaks of the curves at around age 0 (birth), 1–3.5, 6, 8–11, and ≥15 indicated enrichment of cord metabolites significantly associated with BMI at certain ages, implying that these might be potential critical time points for children’s growth. Meanwhile, Figure 4B shows the number of metabolites whose effects on growth outcomes were significantly different between sex (metabolite-by-sex interaction term FDR ≤ 0.05). The absence of peak implied that there was little effect modification by sex on metabolites’ effects on BMI, which was consistent with the sensitivity analysis for longitudinal trajectory analysis (Results Section 2.3 above).

**Figure 5:**
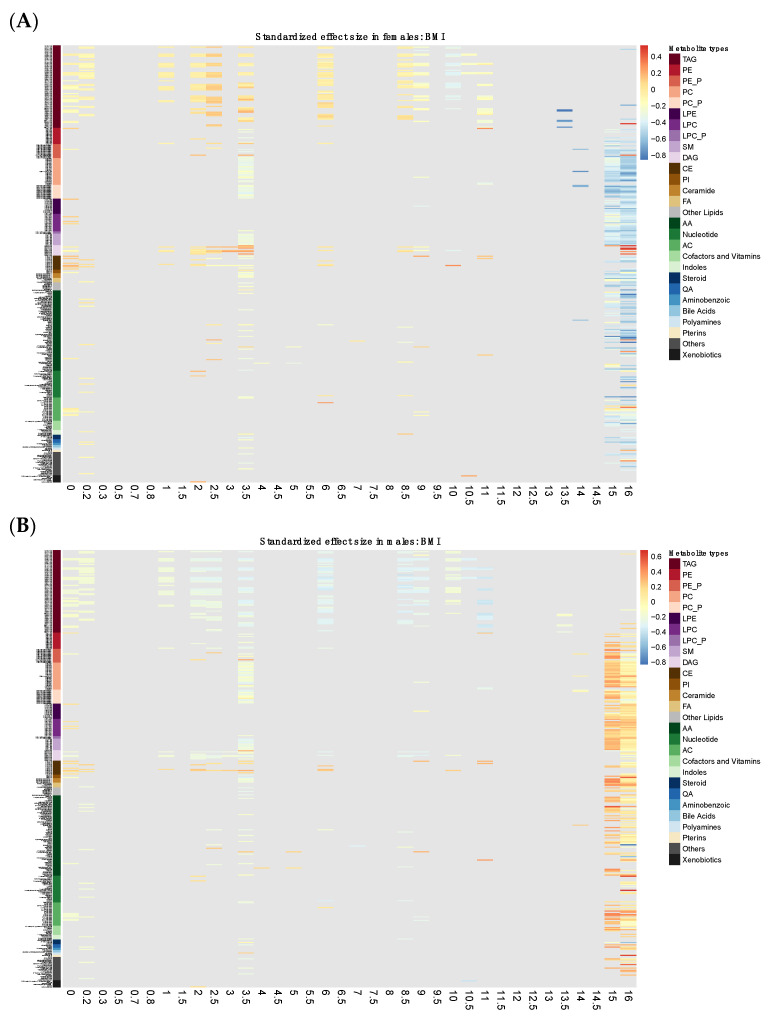
Time-window specific analysis: Heatmap of cord metabolites’ effect size for females (**A**) and males (**B**) on BMI masked by non-significance. The vertical axis represents the 376 metabolites which are ordered based on metabolite types. Effect sizes are standardized by dividing the beta coefficient by the standard deviation of BMI for that sex at that time-window. The color scheme is continuous such that red and blue indicate positive and negative association, respectively. The intensity of the color represents the magnitude of the association. The grey color indicates where the overall effect of that metabolite at that time-window is not statistically significant (LRT FDR > 0.05).

Heatmaps in Figure 5 display the standardized effect size for each metabolite along the 36 time-windows for males and females, respectively, while Supplementary Table S7 lists all the model results. At around birth, ages 1–3.5, and 8–11, TAGs, DAGs, and CEs showed strong correlations with BMI, corroborating the results we saw in the longitudinal trajectory analysis. Apart from this, ACs and some lipid metabolites (PIs and PE_Ps) had negative associations with BMI around birth while some LPCs and LPEs had positive associations with BMI at this time-window. Other lipid metabolites such as PCs, PC_Ps, and SMs appeared to be negatively correlated with BMI at age around 3.5. However, at later ages after 14, TAGs were no longer associated with BMI but associations between most other types of metabolites and BMI showed up to be significant.

### 2.5. Time-Window Specific Analysis: Metabolite Modules

Within every time-window, we fit multivariate linear regressions to test the associations between each of the 7 cord metabolite modules and children’s BMI in that time-window. Model results are listed in Supplementary Table S8. Top metabolite modules were red (n = 25: 20 TAGs, 3 CEs, 1 DAG, and 1 PC; FDR ≤ 0.05 in 13 time-windows), black (n = 23: 22 TAGs and 1 PI; FDR ≤ 0.05 in 11 time-windows), and brown (n = 43: 28 TAGs, 7 DAGs, 7 CEs, and 1 PE; FDR ≤ 0.05 in 10 time-windows).

### 2.6. Time-Window Specific Analysis: Sensitivity Analyses

We repeated the time-window specific analysis for individual metabolites by further adjusting for cesarean section, with the models’ results summarized in Supplementary Table S9. Supplementary Figure S5 shows the number of metabolites that were significantly associated with BMI (panel A) or had significant metabolite-by-sex interaction term (panel B) along time. Since Supplementary Figure S5 was very similar to our main analysis (Figure 4), we concluded that cesarean section did not confound the relationship between cord metabolomics and BMI in any time-window.

In addition, we repeated the time-window specific analysis for individual metabolites by further adjusting for breastfeeding, with the models’ results summarized in Supplementary Table S10. Supplementary Figure S6 shows the number of metabolites that were significantly associated with BMI (panel A) or had significant metabolite-by-sex interaction term (panel B) along time. Since Supplementary Figure S6 was very similar to our main analysis (Figure 4), we concluded that breastfeeding did not confound the relationship between cord metabolomics and BMI in any time-window.

Furthermore, we repeated the time-window specific analysis for individual metabolites by further adjusting for birthweight, with the models’ results summarized in Supplementary Table S11. Compared to the main analysis (Figure 4), Supplementary Figure S7 shows that the associations between cord metabolites and children’s BMI were substantially attenuated by the adjustment of birthweight. In particular, the associations at ages right after birth (0–2) disappeared completely; and much fewer metabolites still had significant associations with BMI at early ages (2–4). According to the heatmaps showing the effect size from these models (Supplementary Figure S8 for this sensitivity analysis as compared to Figure 5 for the main analysis), associations between TAGs, PCs, PC_Ps, and BMI were no longer significant after adjusting for birthweight.

## 3. Discussion

To the best of our knowledge, this is the first large scale metabolomic study in a prospective cohort of US urban low-income underrepresented population, using the cutting-edge liquid chromatography–tandem mass spectrometry (LC-MS). This is also the first study to link cord plasma metabolites with children’s longitudinal BMI trajectories from birth up to age 18 years. This study has made following new contributions to the field.

This study has identified children’s longitudinal BMI trajectories based on high-dimensional clustering, which allows us to gain a better understanding of the role of cord metabolic biomarkers in relation to BMI trajectories. Using k-means clustering and PCA, our analysis revealed four BMI trajectory groups from age 2 to 18 as outcomes (Figure 1B); and using the cutting-edge LC-MS, we profiled 376 metabolites in cord plasma samples as predictors for the BMI trajectories (Figure 2 and Supplementary Table S1). Our exploratory analysis (Figure 2) led us to collapse the four groups into three clinically meaningful groups of children as outcomes. Given the recognition that metabolites in the same or related pathways tend to be correlated, we identified seven metabolite modules among the 376 metabolites (Supplementary Table S2). Two metabolite modules (red and brown) showed significant associations with the three defined BMI trajectory groups (Table 2). Focusing on the 68 metabolites within the two metabolite modules, 27 of them had significant impacts on BMI when comparing early-OWO to NW but very few (only 2) had significant effects when comparing late-OWO to NW (Figure 3 and Supplementary Table S3). However, it was noteworthy that the directions of these associations were consistent across the two comparisons for each metabolite module or individual metabolite. These results suggest that cord plasma metabolites can be most useful in identifying children at risk of early-OWO while they may also hold potential in identifying children with late-OWO.

Studies have shown ample evidence of the persistence of childhood OWO into adulthood [2,3]. Both early and late onset childhood obesity tracks over time and increases the risk of cardiovascular or metabolic disease and cancer in adulthood [14]. Various early school-based and home-based interventions have proven to be effective in preventing childhood obesity to some extent [4]. Therefore, to improve our ability to prevent obesity, it is crucial to identify children at risk of OWO and to intervene at the earliest possible developmental windows. Overall, our study demonstrated the potential of cord metabolite signatures to serve as biomarkers of early onset childhood OWO.

Cord plasma metabolites identified in the longitudinal trajectory analysis were mostly TAGs, DAGs, and CEs which also showed strong correlations with BMI during multiple time-windows in the time-window specific analysis. These findings were supported by existing literatures and biologically plausible as discussed below. As shown in both Figure 3 (longitudinal trajectory analysis) and Figure 5 (time-window specific analysis), the intensities of cord plasma CEs were positively correlated with BMI. Several of the top metabolites were C16:1 CE, C18:3 CE, and C20:3 CE, all with significant associations with BMI when comparing the early-OWO group with the NW group (Figure 3). This finding was consistent with a previous study by Warensjo et al. in which they observed a positive association between the intensities of these CEs in serum and BMI in adulthood (with a mean age of 40) [15].

Our longitudinal trajectory analysis also revealed that the intensities of TAGs and DAGs in cord plasma were negatively correlated with BMI (the associations were significant especially when comparing early-OWO with NW) (Figure 3). The top associations included C52:6 TAG, C54:6 TAG, C52:3 TAG, C54:5 TAG, C36:4 DAG, and C36:3 DAG. Associations between TAGs and BMI showed directions that were consistent with associations between DAGs and BMI, likely because DAGs are precursors in TAG biosynthesis [16]. TAGs are the main source of energy storage specifically in adipose tissues, and thus are expected to be positively related to BMI [17]. However, several previous studies have shown a negative correlation between cord plasma TAG and newborn birthweight [6,18,19,20]. Schaefer-Graf et al. hypothesized that infants with lower birthweight have reduced lipoprotein lipase activity, which results in elevated circulating TAG levels in cord plasma. Consistent with these previous studies, we have demonstrated negative associations between cord plasma TAG and children’s BMI at birth in our time-window specific analysis (Supplementary Table S7); furthermore, we have shown that TAGs were negatively associated with children’s overall longitudinal BMI trajectories (Figure 3). 

In the time-window specific analysis, besides from TAGs, DAGs, and CEs, we have also observed negative correlations between ACs and BMI around birth (Figure 5 and Supplementary Table S7). Some top metabolites were C12:1 carnitine, C14:2 carnitine, C12 carnitine, C10 carnitine, C8 carnitine, and C14:1 carnitine. Previous studies have shown positive associations between serum ACs and BMI in children between 6 and 15 years old and in adults [21,22]. However, we are not aware of studies that have shown any relationships between cord plasma intensities of individual ACs with BMI at birth. Here we proposed a plausible biochemical mechanism of our novel observation. When long-chain fatty acids, such as TAGs measured in this study, are transported into mitochondrial matrix for β-oxidation, ACs are formed from carnitines and acyl-coenzyme A [23]. As our results have shown higher intensities of cord plasma TAGs in children with lower BMI at birth, the translocation of these long-chain TAGs into mitochondria thus produced high levels of ACs in the cord blood plasma.

Moreover, the time-window specific analysis has revealed that there were significant correlations between some lipid metabolites with BMI at birth, but these associations disappeared later and reappeared at ages greater than 15 (Figure 5 and Supplementary Table S7). For example, C18:1 LPC and C16:1 LPC were positively associated with BMI at birth in both sexes, consistent with findings by Lu et al [9]. One plausible explanation for this observed association is hypoxia activating phospholipases that promote synthesis of LPCs. Larger infants (with greater birthweight) experience extended hypoxic periods during prolonged delivery and thus produces elevated LPC levels in cord blood [9]. Evidence for relationships between LPCs and childhood obesity had been contradictive [24,25,26]. Hellmuth et al. reported no association of C18:1 LPC or C16:1 LPC with BMI among children up to 15 years old [27]. Our study had followed subjects to more than 16 years old and showed significant correlations between these LPCs and BMI at age greater than 15, indicating potentials of cord blood LPCs having long term impacts on children’s BMI. However, we need a larger sample size to validate the observed association (current n = 68 at age 15, n = 57 at age > 16) at the last time-window for age greater than 16.

For the longitudinal trajectory analysis, we conducted a sensitivity analysis by including a multiplicative interaction term between sex and metabolite module (or single metabolite) in the multinomial logistic regression models. The results indicated that neither the effects of single metabolites nor the effects of metabolite modules differed significantly between sex (Supplementary Table S5, Supplementary Table S6, Supplementary Figure S2).

For the time-window specific analysis, we conducted three sensitivity analyses by further adjusting for cesarean section, breastfeeding, and birthweight, respectively, in the linear regression models within each time-window. The results showed that cesarean section, which might impact gut microbiome in newborns, did not confound the relationship between cord metabolomics and childhood obesity [28]. Moreover, breastfeeding, a known protective factor against childhood obesity, did not confound the relationship between cord metabolomics and childhood obesity [29]. It was expected that further adjusting for birthweight would attenuate the significant correlations we had observed between metabolites and children’s BMI because birthweight was highly predictive of children’s growth at early ages. However, as various literatures have demonstrated associations between cord metabolic profile and birthweight [6,7,8,9], the temporal nature of this correlation/causation was still unclear as whether birthweight affected cord metabolomics (in which case birthweight would be a confounder that we should adjust for in our models) or cord metabolomics affected birthweight (in which case birthweight should be treated as a mediator in our analysis and thus should not be adjusted in the models). The direction of this correlation would be a challenging question to be answered in future studies, and before that, we wish to steer the focus towards our main analysis which explored the more straightforward relationship between cord metabolites and children’s longitudinal BMI without considering birthweight in the picture.

This study had several strengths. Though previous studies have examined relationships between cord metabolite signatures and birth outcomes as well as childhood obesity risk, to our knowledge this study is the first to explore the relationship between cord blood plasma metabolomics and children’s longitudinal BMI trajectories. This study has used novel approaches to define longitudinal BMI trajectories into clinically meaningful trajectory categories for each child. Furthermore, the study examined not only single metabolites’ effects but also metabolites’ combined effects (as modules) on children’s BMI trajectories by identifying novel metabolic networks based on the metabolomic data. Moreover, our study carried out an additional time-window specific analysis as an alternative approach to this longitudinal data and demonstrated consistent results with our longitudinal trajectory analysis. In addition, this study used a prospective birth cohort study design. With metabolites measured at birth, our finding of the identified metabolite—BMI trajectory associations has a clear temporal relationship and should not be confounded by reverse causation. The BBC is an under-represented, urban, low-income multi-ethnic population. Thus, findings from this study will have direct relevance to understanding and preventing obesity as well as reducing health disparities in this high-risk, predominantly Black and Hispanic population. Our findings remain to be replicated in other independent cohorts with similar and different socio-demographic and clinical characteristics.

This study also had some limitations. In order to conduct k-means clustering and PCA on the longitudinal BMI data, we had to impute missing BMI measurements by averaging the last and next observed values. This imputation might have overlooked variations in children’s BMI in between the two observed time points, which might lead to misclassification of BMI trajectory and attenuation of the observed associations with cord blood metabolites. Moreover, this study did not adjust in our models for additional covariates such as mothers’ nutritional information, children’s physical activities, and family nutrition data during follow-up; and these variables need to be further considered in future studies. Although our study was by far the largest metabolomics study on cord plasma metabolites in a well characterized longitudinal cohort, the sample size was limited at older age groups when participants in the BBC study were still being followed-up. Finally, caution is needed when generalizing our findings to other populations with different social-demographic and clinical characteristics.

Contrary to traditional longitudinal data analysis which usually uses mixed-effects models, our study took a different approach in exploring the longitudinal data by defining longitudinal trajectory or looking at disjoint time-windows separately. Regression models such as mixed-effects models required assuming functional form, often linear relationship of the time effect. However, we wish to avoid assumptions of linear models and to use non-parametric clustering methods to identify growth trajectory patterns. It turned out that some children’s BMI growth trajectory appeared to be non-linear (such as the early-OWO and late-OWO groups). In contrast to the mixed-effects models which might not be able to capture these non-linear patterns, our longitudinal trajectory analysis could distinguish linear or non-linear patterns and revealed clinically meaningful BMI trajectories, which could be leveraged to offer a novel way to elucidate longitudinal associations of cord metabolomics with BMI trajectory from birth to adolescence.

## 4. Materials and Methods

### 4.1. Study Population

This study used data from the Boston Birth Cohort (BBC), a US predominantly urban, low-income multi-ethnic birth cohort that consisted of 3153 mother–infant pairs by 2018. Participants were enrolled at birth and followed prospectively at the Boston Medical Center, Boston, MA. A detailed description of the BBC was previously reported [30]. In brief, rolling enrollment for the BBC began in 1998 for which mothers enrolled within 24–72 h after delivery, provided written consent, and completed a questionnaire interview. Maternal and infant clinical information were obtained from their medical records. Multiple-gestation pregnancies, infants with major birth defects or in vitro fertilized pregnancies, or whose siblings were already enrolled in the study were excluded. This study included 946 (44.8% female) children with data on cord plasma metabolome and BMI measurements after age 2 years. Their perinatal and postnatal variables [31] were summarized by BMI trajectory groups (see Methods Section 4.3.1 and Results Section 2.1) and compared between groups using one-way ANOVA or the Kruskal–Wallis test for continuous variables and Pearson’s chi-squared test for categorical variables (Table 1). The study protocol was approved by the Institutional Review Boards of the Johns Hopkins Bloomberg School of Public Health and Boston Medical Center.

### 4.2. Cord Plasma Metabolites

Umbilical cord blood samples were collected at delivery by trained nursing staff at Boston Medical Center following the guidelines of the National Heart, Lung, and Blood Institute Working Group on Blood Drawing, Processing, and Storage for Genetic Studies [32]. The cord blood plasma samples were stored in −80 °C freezers as aliquots of 150 μL until they were shipped with dry ice to the Metabolite Profiling Laboratory at the Broad Institute of MIT and Harvard for metabolomic profiling. Metabolomics were profiled using C8-pos (reversed-phase C8 chromatography/positive ion mode which detects lipids) and HILIC-pos (hydrophilic interaction chromatography/positive ion mode which detects polar molecules) LC-MS. The LC apparatus was comprised of an Agilent 1200 liquid chromatography series pump (Agilent Technologies, CA, USA) coupled to a CTC-PAL HTS-*xt* autosampler (Leap Technologies, NC, USA), and the MS apparatus was comprised of a 4000 QTRAP triple quadrupole mass spectrometer (AB SCIEX, MA, USA) coupled to an electrospray source (Turbo V from AB SCIEX, MA, USA) [33]. Along with 190 μL of isopropanol containing an internal standard of 1-dodecanoyl-2-tridecanoyl-sn-glycero-3-phosphocholine, 10 μL of cord plasma were extracted and then centrifuged such that the supernatants were injected directly. Automated peak integration was carried out using MultiQuant software and MS analyses were conducted using electrospray ionization and Q1 scans in the positive-ion mode [34]. After excluding 19 metabolites with coefficients of variation greater than 20% and comparing to library entries of purified known standards, 376 metabolites were available for this analysis. Non-detectable intensities were imputed as half of the minimum intensity of the metabolite. To reduce bias due to outliers and skewed distribution, inverse normal transformation was performed on each metabolite for subsequent analysis.

### 4.3. Statistical Analyses

#### 4.3.1. Longitudinal Trajectory Analysis: Categorizing Longitudinal BMI Trajectories

Children’s repeated measurements of BMI from birth to age 18 were divided into 36 time-windows based on available samples for different ages groups such that each time-window had measurements from at least 30 participants and the window length was no longer than 12 months. BMIPCT) at each measurement was calculated based on U.S. national reference data by age and sex [35] (available only for age ≥2 years old) and then averaged within each time-window, resulting in BMIPCT from age 2 to age 18 in 28 time-windows. Missing BMIPCT were imputed using the average of last and next observed values. Data can be unavailable either due to children not reaching that age or missing some visits. We applied k-means clustering to the BMIPCT-by-time-window matrix to cluster children, with k chosen to be 2 which maximized the group distinction. Next, participants in each cluster were further divided into two groups based on PCA of the BMIPCT-by-time-window matrix, resulting in four groups of children. Figure 1B illustrates children’s individual longitudinal BMIPCT trajectories as well as a LOWESS (locally weighted scatterplot smoothing) smoothing curve for each of the four groups. We named these four groups of children based on the smoothing curves of BMI trajectories (shown in Results Section 2.1 and Figure 1B) as early onset overweight or obesity (early-OWO), late onset overweight or obesity (late-OWO), normal weight trajectory A (NW-A) and normal weight trajectory B (NW-B).

Characteristics of the four groups of children were summarized and compared in Table 1. As an exploratory analysis, we fit multinomial logistic regression models of the four groups on each metabolite respectively, using NW-A as the reference group. To visualize the impact of individual metabolites on each of the three comparisons made in the regression, we used the pheatmap function in R to construct heatmaps of the 376 metabolites’ effect size for each comparison. Metabolites were ordered by types, with the 194 lipid metabolites measured by C8-pos first and then the 182 metabolites measured by HILIC-pos, as shown in the rainbow legend in the heatmaps (Figure 2, Figure 5, Supplementary Figure S2 and Supplementary Figure S4). Colors in the heatmaps indicated the direction and magnitude of the effect size. The heatmaps were masked in two ways: (1) for the first 3 columns, metabolites with FDR > 0.05 were shown in grey; (2) for the last 3 columns, metabolites with unadjusted *p*-value > 0.05 were shown in grey. Through this exploratory analysis, our goal was to explore if any difference is detectable between each group and the reference group; if not, then we would consider combining that certain group with the reference group to achieve a more succinct characterization of children’s longitudinal BMI trajectories. According to the heatmaps (shown in Results Section 2.1 and Figure 2), the NW-A and NW-B groups were combined into one group: normal weight trajectory (NW).

#### 4.3.2. Longitudinal Trajectory Analysis: Metabolite Modules and BMI Trajectory Association

To study metabolites’ combined effects on longitudinal BMI trajectories, we used the WGCNA package [13] to identify metabolite network modules based on correlation between metabolite pairs, setting minimum module size as 15 and power as 7 for which the scale-free topology fit index reached a plateau at a high value (roughly 0.80). Each module was assigned a color (Supplementary Table S2) and represented by the PC1 of member metabolites as a manifestation of that module’s relative intensity in cord plasma. To test for association with BMI trajectories, we fit multinomial logistic regression models of the three trajectory groups (early-OWO, late-OWO vs. NW as the reference group) on PC1 of each module, respectively. We followed up the metabolite modules with significant associations with BMI trajectory (*p*-value ≤ 0.05) and tested associations between individual metabolites in these modules with the three BMI trajectory groups.

#### 4.3.3. Longitudinal Trajectory Analysis: Sensitivity Analysis

To explore whether the metabolites’ effects differed by sex, we repeated the above longitudinal trajectory analyses but included a multiplicative interaction term between sex and metabolite module (or single metabolite) in the logistic regression models. A likelihood ratio test (LRT) was applied to test the overall effect of each metabolite module (or single metabolite) including the main effect and the interaction with sex.

#### 4.3.4. Time-window Specific Analysis: Individual Metabolites

As described in Methods Section 4.3.1, we have divided each child’s repeated measurements of BMI from birth to adolescence into 36 disjoint time-windows. For each time-window, we fit multiple linear regression of average BMI on individual cord plasma metabolite adjusting for subjects’ mean age in that time-window. We tested the effect modification by sex on metabolites’ effects through including a multiplicative interaction term between sex and metabolite levels in the model. The overall effect of each metabolite, consisting of both the main effect and its interaction with sex, was evaluated by a likelihood ratio test (LRT). As the variance of BMI changed with ages, in order to compare metabolites’ effects across ages, the effect size of each metabolite in each sex was standardized by dividing the beta coefficient from the regression model by the standard deviation of BMI at the corresponding time-window for that sex group.

Similar to Methods Section 4.3.1, we constructed heatmaps of the 376 metabolites’ effect size for all 36 time-windows (Figure 5, Supplementary Figure S4). While the colors in each cell of the heatmaps represented the direction and magnitude of the standardized effect size, they were masked in two ways: (1) in Figure 5, metabolites with LRT FDR > 0.05 were shown in grey; (2) in Supplementary Figure S4, metabolites with sex and metabolite interaction term FDR > 0.05 were shown in grey. We also provided scatter plots of standardized effect size in females against males in Supplementary Figure S3 so as to compare metabolites’ effects in the two sex groups. The scatter plots were divided in 36 panels for the 36 time-windows.

#### 4.3.5. Time-Window Specific Analysis: Metabolite Modules

As explained in Methods Section 4.3.2, we have identified metabolite modules based on correlation between metabolite pairs. To study metabolites’ combined effects on BMI, within each time-window we performed multiple linear regression of BMI on each metabolite module’s PC1 adjusting for age in that time-window, and with a multiplicative interaction term between the module and sex. Likewise, we standardized each metabolite module’s effect size by time-window, and examined each module’s overall effect through the LRT as well as the effect modification by sex via the interaction term.

#### 4.3.6. Time-Window Specific Analysis: Sensitivity Analyses

Studies have shown that cesarean section had short-term risks such as altered gut microbiome in newborns which had an impact on childhood obesity [28]. To explore the role of cesarean section in childhood obesity, we repeated the time-window specific analysis for individual metabolites (Methods Section 4.3.4) by further adjusting each linear regression model for cesarean section.

In addition, breastfeeding has been identified as a protective factor against childhood obesity [29]. Therefore, we conducted another sensitivity analysis by repeating the time-window specific analysis for individual metabolites (Methods Section 4.3.4) while further adjusting each linear regression model for breastfeeding.

Furthermore, as birthweight has been shown to be associated with both cord metabolomics and increased adiposity in childhood [6,7,8,9,36], we repeated the time-window specific analysis for individual metabolites (Methods Section 4.3.4) once again by further adjusting each linear regression model for birthweight in all time-windows except for the first time-window at birth.

For all analyses, models were adjusted for sex, race, preterm birth, maternal smoking during pregnancy, maternal BMI and maternal education; multiple hypothesis testing was corrected using the false discovery rate (FDR) for the number of metabolites (or metabolite modules) tested within each regression model. We used an α-level of 0.05 as the significance threshold. Data were analyzed using RStudio Version 1.2.1335.

## 5. Conclusions

In this prospective birth cohort, we demonstrated distinctive BMI trajectories from birth to adolescence. We identified individual cord plasma metabolites and network modules that were significantly associated with early onset OWO that persisted into adolescence. Furthermore, we discovered several critical time-windows when cord metabolites were strongly associated with BMI. The findings, if further confirmed, could shed new light on biological pathways underlying early onset/persistent OWO and guide early interventions to mitigate or reverse its BMI trajectory and associated long-term impacts.

## Figures and Tables

**Table 1:** Characteristics of mother–child pairs stratified by children’s BMI trajectory groups ^a^.

	Early-OWO ^a^	Late-OWO ^a^	NW-A ^a^	NW-B ^a^	*p*-Value
N	388	254	186	118	
**Maternal Characteristics**					
Age of delivery (years)	28.7 ± 6.7	29.0 ± 6.8	27.4 ± 6.5	27.7 ± 6.3	0.038
Race/ethnicity					0.030
Black	230 (59.3%)	162 (63.8%)	90 (48.4%)	74 (62.7%)	
Hispanic	95 (24.5%)	49 (19.3%)	49 (26.3%)	30 (25.4%)	
White	21 (5.4%)	13 (5.1%)	12 (6.5%)	2 (1.7%)	
Others	42 (10.8%)	30 (11.8%)	35 (18.8%)	12 (10.2%)	
Education					0.291
High school and above	262 (67.5%)	187 (73.6%)	140 (75.3%)	88 (74.6%)	
Less than high school	123 (31.7%)	65 (25.6%)	46 (24.7%)	30 (25.4%)	
Unknown	3 (0.8%)	2 (0.8%)	0 (0.0%)	0 (0.0%)	
Smoking during pregnancy					0.112
Continuous	52 (13.4%)	21 (8.3%)	14 (7.5%)	9 (7.6%)	
Intermittent	24 (6.2%)	19 (7.5%)	14 (7.5%)	9 (7.6%)	
Never	311 (80.2%)	208 (81.9%)	156 (83.9%)	99 (83.9%)	
Unknown	1 (0.3%)	6 (2.4%)	2 (1.1%)	1 (0.8%)	
Maternal pregnancy overweight or obesity					<0.001
No	151 (38.9%)	109 (42.9%)	109 (58.6%)	61 (51.7%)	
Yes	216 (55.7%)	136 (53.5%)	63 (33.9%)	50 (42.4%)	
Unknown	21 (5.4%)	9 (3.5%)	14 (7.5%)	7 (5.9%)	
Cesarean section (N = 945)	134 (34.6%)	103 (40.6%)	58 (31.2%)	21 (17.8%)	<0.001
Breastfeeding (N = 901)					0.029
Both bottle-fed and breast-fed	243 (65.5%)	153 (62.7%)	118 (69.4%)	85 (73.3%)	
Bottle-fed	107 (28.8%)	68 (27.9%)	33 (19.4%)	21 (18.1%)	
Breast-fed	21 (5.7%)	23 (9.4%)	19 (11.2%)	10 (8.6%)	
**Child’s Characteristics**					
Sex: females	173 (44.6%)	121 (47.6%)	76 (40.9%)	54 (45.8%)	0.563
Birthweight (g)	3229.6 ± 678.4	3068.2 ± 675.2	3009.8 ± 641.1	2837.6 ± 650.7	<0.001
Gestational age (weeks)	38.6 ± 2.4	38.4 ± 2.6	38.7 ± 2.5	38.3 ± 2.7	0.340
Preterm	75 (19.3%)	45 (17.7%)	26 (14.0%)	23 (19.5%)	0.440
Parity					0.205
0	163 (42.0%)	113 (44.5%)	90 (48.4%)	38 (32.2%)	
1	110 (28.4%)	63 (24.8%)	48 (25.8%)	40 (33.9%)	
2	59 (15.2%)	44 (17.3%)	32 (17.2%)	23 (19.5%)	
3+	56 (14.4%)	34 (13.4%)	16 (8.6%)	17 (14.4%)	
Age at last visit (years) ^b^	9.2 (6.3–12.2)	9.2 (7.1–11.1)	7.8 (5.1–10.3)	9.8 (7.2–12.7)	<0.001
Height at last visit (cm) ^b^	138.9 (120.5–156.1)	139.2 (122.7–150.9)	126.2 (109.5–140.0)	135.6 (122.4–155.9)	<0.001
Weight at last visit (kg) ^b^	40.9 (27.3–60.8)	40.7 (27.3–55.0)	23.7 (17.1–31.5)	30.2 (23.2–44.6)	<0.001
BMI at last visit (kg/cm^2^) ^b^	21.3 (17.9–25.5)	21.2 (17.8–24.5)	15.4 (14.6–16.0)	16.2 (15.4–18.2)	<0.001
Overweight or obesity at last visit	252 (64.9%)	170 (66.9%)	0 (0.0%)	3 (2.5%)	<0.001

Data are presented as either mean ± SD or no. (%); *p* values are from one-way ANOVA or chi-squared test. ^a^ Grouping of children is based on their longitudinal BMI trajectories (explained in Methods and Results): early onset overweight and obesity (early-OWO), late onset OWO (late-OWO), normal weight trajectory A (NW-A), normal weight trajectory B (NW-B) ^b^ Data are presented as median (IQR); *p* values are from Kruskal-Wallis test.

**Table 2:** Longitudinal trajectory analysis: Associations of cord metabolite modules (as defined based on correlation between metabolite pairs) with child BMI trajectory groups (early-OWO and late-OWO as compared to NW) from multinomial logistic regression model results.

Metabolite Module	Early-OWO vs. NW			Late-OWO vs. NW		
	Odds Ratio (95% CI)	*p*-Value	FDR ^a, b^	Odds Ratio (95% CI)	*p*-Value	FDR ^b^
red	0.95 (0.91, 0.99)	0.006	0.043	0.96 (0.92, 1.00)	0.045	0.159
brown	0.96 (0.93, 0.99)	0.014	0.049	0.96 (0.93, 1.00)	0.038	0.159
black	0.97 (0.93, 1.01)	0.163	0.352	0.96 (0.92, 1.00)	0.069	0.161
green	1.02 (0.99, 1.06)	0.201	0.352	1.01 (0.98, 1.05)	0.481	0.728
yellow	1.02 (0.98, 1.06)	0.275	0.386	1.00 (0.97, 1.05)	0.808	0.808
blue	1.00 (0.98, 1.02)	0.924	0.924	1.01 (0.98, 1.03)	0.520	0.728
turquoise	1.00 (0.98, 1.02)	0.829	0.924	1.01 (0.98, 1.03)	0.685	0.799

^a^ Rows (metabolite modules) are ordered based on FDR of early-OWO vs. NW comparison. ^b^ FDR accounts for multiple hypothesis testing across all 7 metabolite modules.

## Data Availability

Data described in the manuscript, code book, and analytic code will be made available upon request pending review and approval by the Institutional Review Board.

## Supplementary Materials

The following are available online at https://www.mdpi.com/article/10.3390/metabo11110739/s1, Figure S1: Longitudinal trajectory analysis: Plot of PC2 vs. PC1 for 946 subjects colored by k-means clustering result, Figure S2: Longitudinal trajectory analysis—sensitivity analysis: Heatmap of the effect size of metabolites on BMI trajectory (4 groups) for females (first 3 columns) and males (last 3 columns) respectively on the horizontal axis, Figure S3: Time-window specific analysis: Scatter plot of the standardized effect size in males vs. females for BMI, Figure S4: Time-window specific analysis: Heatmap of cord metabolites’ effect size for females (Panel A) and males (Panel B) on BMI masked by non-significance of interaction term FDR, Figure S5: Time-window specific analysis—sensitivity analysis further adjusted for cesarean section: Number of significant metabolites at each time-window based on LRT FDR (Panel A), number of metabolites that have significantly different effects between two sexes at each time-window based on metabolite-by-sex interaction term FDR (Panel B), Figure S6: Time-window specific analysis—sensitivity analysis further adjusted for breastfeeding: Number of significant metabolites at each time-window based on LRT FDR (Panel A), number of metabolites that have significantly different effects between two sexes at each time-window based on metabolite-by-sex interaction term FDR (Panel B), Figure S7: Time-window specific analysis—sensitivity analysis further adjusted for birthweight: Number of significant metabolites at each time-window based on LRT FDR (Panel A), number of metabolites that have significantly different effects between two sexes at each time-window based on metabolite-by-sex interaction term FDR (Panel B), Figure S8: Time-window specific analysis—sensitivity analysis further adjusted for birthweight: Heatmap of cord metabolites’ effect size for females (Panel A) and males (Panel B) on BMI masked by non-significance, Table S1: Longitudinal trajectory analysis: Associations of all 376 individual cord metabolites with child BMI trajectory groups (early-OWO, late-OWO and NW-B as compared to NW-A) from multinomial logistic regression model results, Table S2: Longitudinal trajectory analysis: Color assignment for cord metabolites modules, Table S3: Longitudinal trajectory analysis: Associations of individual cord metabolites within each significant module (as identified in Table 2 with *p*-value ≤ 0.05 for either comparison) with child BMI trajectory groups (early-OWO and late-OWO as compared to NW) from multinomial logistic regression model results, Table S4: Longitudinal trajectory analysis: Associations of all 376 individual cord metabolites with child BMI trajectory groups (early-OWO and late-OWO as compared to NW-A) from multinomial logistic regression model results, Table S5: Longitudinal trajectory analysis—sensitivity analysis: Associations of cord metabolite modules (as defined based on correlation between metabolite pairs) with child BMI trajectory groups (early-OWO and late-OWO as compared to NW) from multinomial logistic regression model results with interaction term between sex and metabolite modules, Table S6: Longitudinal trajectory analysis—sensitivity analysis: Associations of individual cord metabolites within each significant module (as identified in Table 2 with *p*-value ≤ 0.05 for either comparison) with child BMI trajectory groups (early-OWO and late-OWO as compared to NW) from multinomial logistic regression model results with an interaction term between sex and metabolite intensity, Table S7: Time-window specific analysis: Summary of time-window specific linear regression models’ results for BMI, Table S8: Time-window specific analysis: Summary of time-window specific linear regression models’ results for BMI, Table S9: Time-window specific analysis - sensitivity analysis further adjusted for cesarean section: Summary of time-window specific linear regression models’ results for BMI, Table S10: Time-window specific analysis—sensitivity analysis further adjusted for breastfeeding: Summary of time-window specific linear regression models’ results for BMI, Table S11: Time-window specific analysis—sensitivity analysis further adjusted for birthweight: Summary of time-window specific linear regression models’ results for BMI.

## Abbreviations

TAG (triacylglycerol); PE (phosphatidylethanolamine); PE_P (phosphatidylethanolamine plasmalogen); PC (phosphatidylcholine); PC_P (phosphatidylcholine plasmalogen); LPE (lysophosphatidylethanolamine); LPC (lysophosphatidylcholine); LPC_P (lysophosphatidylcholine plasmalogen); SM (sphingomyelin); DAG (diacylglycerol); CE (cholesterol ester); PI (phosphatidylinositol); FA (fatty acids); AA (amino acids); AC (acylcarnitine); QA (quaternary amines).
