# Supplementary material for: Cord Blood Metabolome and BMI Trajectory from Birth to Adolescence: A Prospective Birth Cohort Study on Early Life Biomarkers of Persistent Obesity
Source: Metabolites. 2021 Oct 28;11(11):739. doi: 10.3390/metabo11110739 (PMC8625562; doi:10.3390/metabo11110739)
Supplement: Supplementary file 1 [file metabolites-11-00739-s001.zip › metabolites-1402936-supplementary/Supplementary Tables and figures.pdf]

Supplementary Table S1. Longitudinal trajectory analysis: Associations of all 376 individual cord metabolites with child BMI trajectory groups (early-OWO, late-OWO and NW-B as compared to NW-A) from multinomial logistic regression model results.

(Table attached as one Excel file)

*Rows are metabolites ordered based on FDR of Early-OWO vs. NW-A comparison.

Supplementary Table S2. Longitudinal trajectory analysis: Color assignment for cord metabolites modules. Hierarchical clustering and WGCNA package were applied to group cord metabolites based on correlation between metabolite pairs and assigned colors to each module. The 376 metabolites were ordered in the same way as they were in the heatmaps.

(Table attached as one Excel file)

*Metabolites within the grey module are considered as those that are not assigned to a certain module by the WGCNA package, and thus excluded from the analysis in Table 2.

Supplementary Table S3. Longitudinal trajectory analysis: Associations of individual cord metabolites within each significant module (as identified in Table 2 with p-value≤0.05 for either comparison) with child BMI trajectory groups (early-OWO and late-OWO as compared to NW) from multinomial logistic regression model results. All 68 metabolites in the 2 metabolite modules identified in Table 2 are shown here.

(Table attached as one Excel file)

*Rows (metabolites) are ordered based on FDR of Early-OWO vs. NW comparison.
*FDR accounts for multiple hypothesis testing across all 68 metabolites in the 2 candidate metabolite modules identified in Table 2.

Supplementary Table S4. Longitudinal trajectory analysis: Associations of all 376 individual cord metabolites with child BMI trajectory groups (early-OWO and late-OWO as compared to NW-A) from multinomial logistic regression model results.

(Table attached as one Excel file)

*Rows (metabolites) are ordered based on FDR of Early-OWO vs. NW comparison.

Supplementary Table S5. Longitudinal trajectory analysis - sensitivity analysis: Associations of cord metabolite modules (as defined based on correlation between metabolite pairs) with child BMI trajectory groups (early-OWO and late-OWO as compared to NW) from multinomial logistic regression model results with interaction term between sex and metabolite modules.

Metabolite module	Early-OWO vs. NW			Late-OWO vs. NW			LRT p-value	LRT FDR[b]
	Odds ratio (95% CI)	Interaction term p-value	Interaction term FDR[b]	Odds ratio (95% CI)	Interaction term p-value	Interaction term FDR[b]		
brown	0.98 (0.94, 1.03)	0.189	0.499	0.94 (0.90, 0.99)	0.225	0.529	0.009	0.06
red	0.97 (0.92, 1.03)	0.214	0.499	0.96 (0.91, 1.02)	0.798	0.798	0.044	0.152
black	0.98 (0.92, 1.04)	0.74	0.86	0.93 (0.87, 1.00)	0.227	0.529	0.175	0.359
green	0.99 (0.95, 1.05)	0.137	0.499	1.02 (0.97, 1.08)	0.631	0.749	0.205	0.359
blue	0.99 (0.95, 1.02)	0.334	0.585	1.01 (0.98, 1.05)	0.642	0.749	0.592	0.719
turquoise	1.01 (0.97, 1.04)	0.782	0.86	1.03 (0.99, 1.06)	0.14	0.529	0.616	0.719
yellow	1.02 (0.97, 1.08)	0.86	0.86	1.02 (0.96, 1.08)	0.555	0.749	0.793	0.793

[a]Rows (metabolite modules) are ordered based on FDR for the likelihood ratio test.
[b]FDR accounts for multiple hypothesis testing across all 6 metabolite modules.

Supplementary Table S6. Longitudinal trajectory analysis - sensitivity analysis: Associations of individual cord metabolites within each significant module (as identified in Table 2 with p-value≤0.05 for either comparison) with child BMI trajectory groups (early-OWO and late-OWO as compared to NW) from multinomial logistic regression model results with an interaction term between sex and metabolite intensity. All 68 metabolites in the 2 metabolite modules identified in Supplementary Table 5 are shown here.

(Table attached as one Excel file)

*Rows (metabolite) are ordered based on FDR for the likelihood ratio test.
*FDR accounts for multiple hypothesis testing across all 68 metabolites in the 2 candidate metabolite modules identified in Supplementary Table 5.

Supplementary Table S7. Time-window specific analysis: Summary of time-window specific linear regression models' results for BMI. Rows are 376 cord metabolites ordered by metabolite types and columns are metabolites' standardized effect size for females and males, FDR for the sex*metabolite interaction term, and FDR for the LRT from the models for each of the 36 time-windows.

(Table attached as one Excel file)

Supplementary Table S8. Time-window specific analysis: Summary of time-window specific linear regression models' results for BMI. Rows are 7 metabolite modules and columns are metabolite modules' standardized effect size for females and males, FDR for the sex*module interaction term, and FDR for the LRT from the models for each of the 36 time-windows.

(Table attached as one Excel file)

Supplementary Table S9. Time-window specific analysis - sensitivity analysis further adjusted for cesarean section: Summary of time-window specific linear regression models' results for BMI. Rows are 376 cord metabolites ordered by metabolite types and columns are metabolites' standardized effect size for females and males, FDR for the sex*metabolite interaction term, and FDR for the LRT from the models for each of the 36 time-windows.

(Table attached as one Excel file)

Supplementary Table S10. Time-window specific analysis - sensitivity analysis further adjusted for breastfeeding: Summary of time-window specific linear regression models' results for BMI. Rows are 376 cord metabolites ordered by metabolite types and columns are metabolites' standardized effect size for females and males, FDR for the sex*metabolite interaction term, and FDR for the LRT from the models for each of the 36 time-windows.

(Table attached as one Excel file)

Supplementary Table S11. Time-window specific analysis - sensitivity analysis further adjusted for birthweight: Summary of time-window specific linear regression models' results for BMI. Rows are 376 cord metabolites ordered by metabolite types and columns are metabolites' standardized effect size for females and males, FDR for the sex*metabolite interaction term, and FDR for the LRT from the models for each of the 36 time-windows.

(Table attached as one Excel file)

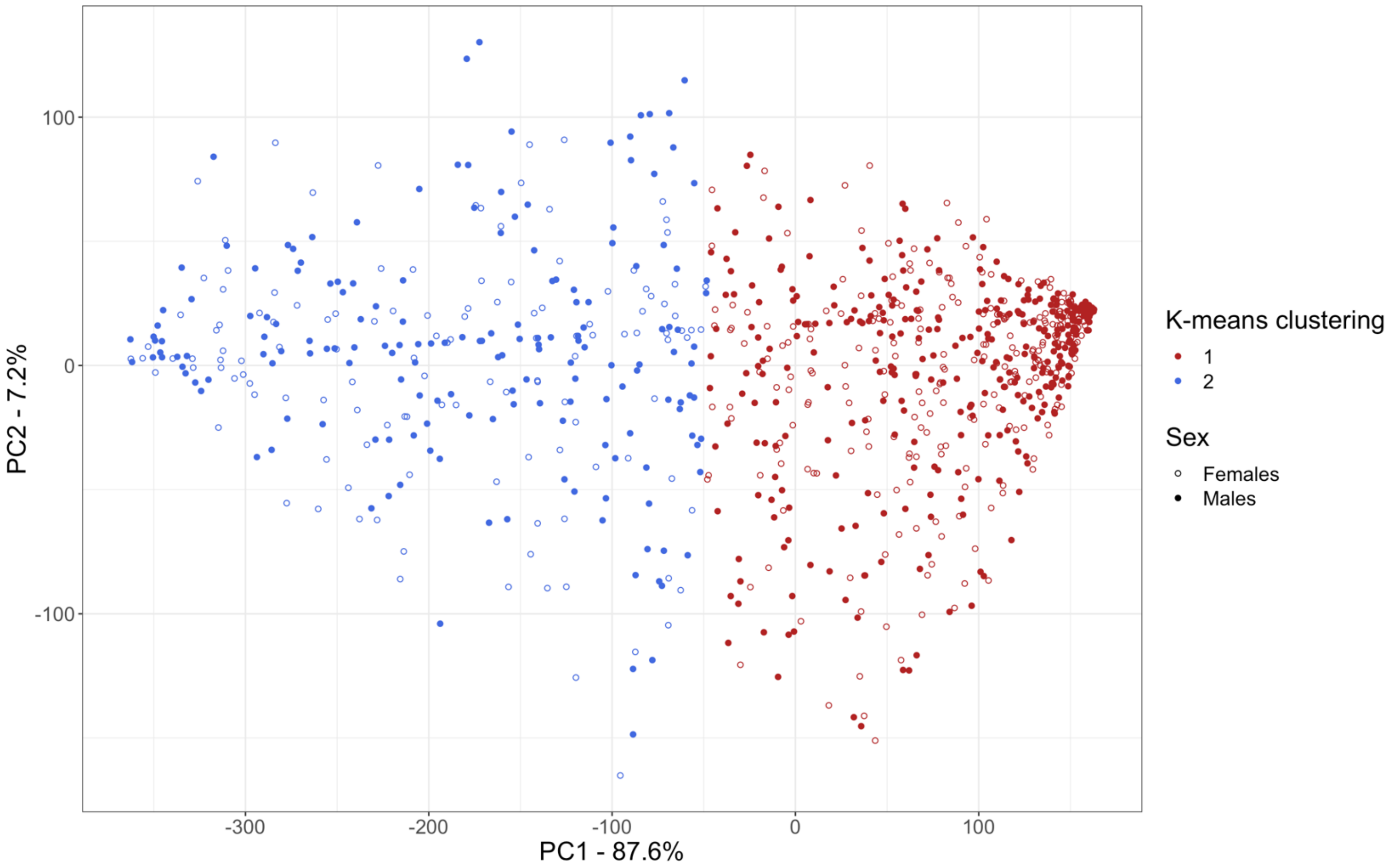

Supplementary Figure S1. Longitudinal trajectory analysis: Plot of PC2 vs. PC1 for 946 subjects colored by k-means clustering result. Children in cluster 1 (n = 642) are separated from children in cluster 2 (n = 304) based on PC1. Zero value of PC2 is used to further divide children into 4 groups: K-means cluster 1 and positive PC2 (n=388) group denoted as "early-OWO", K-means cluster 1 + negative PC2 (n=254) group denoted as "late-OWO", K-means cluster 2 and positive PC2 (n=186) group denoted as "NW-A", and K-means cluster 2 and negative PC2 (n=118) group denoted as "NW-B".

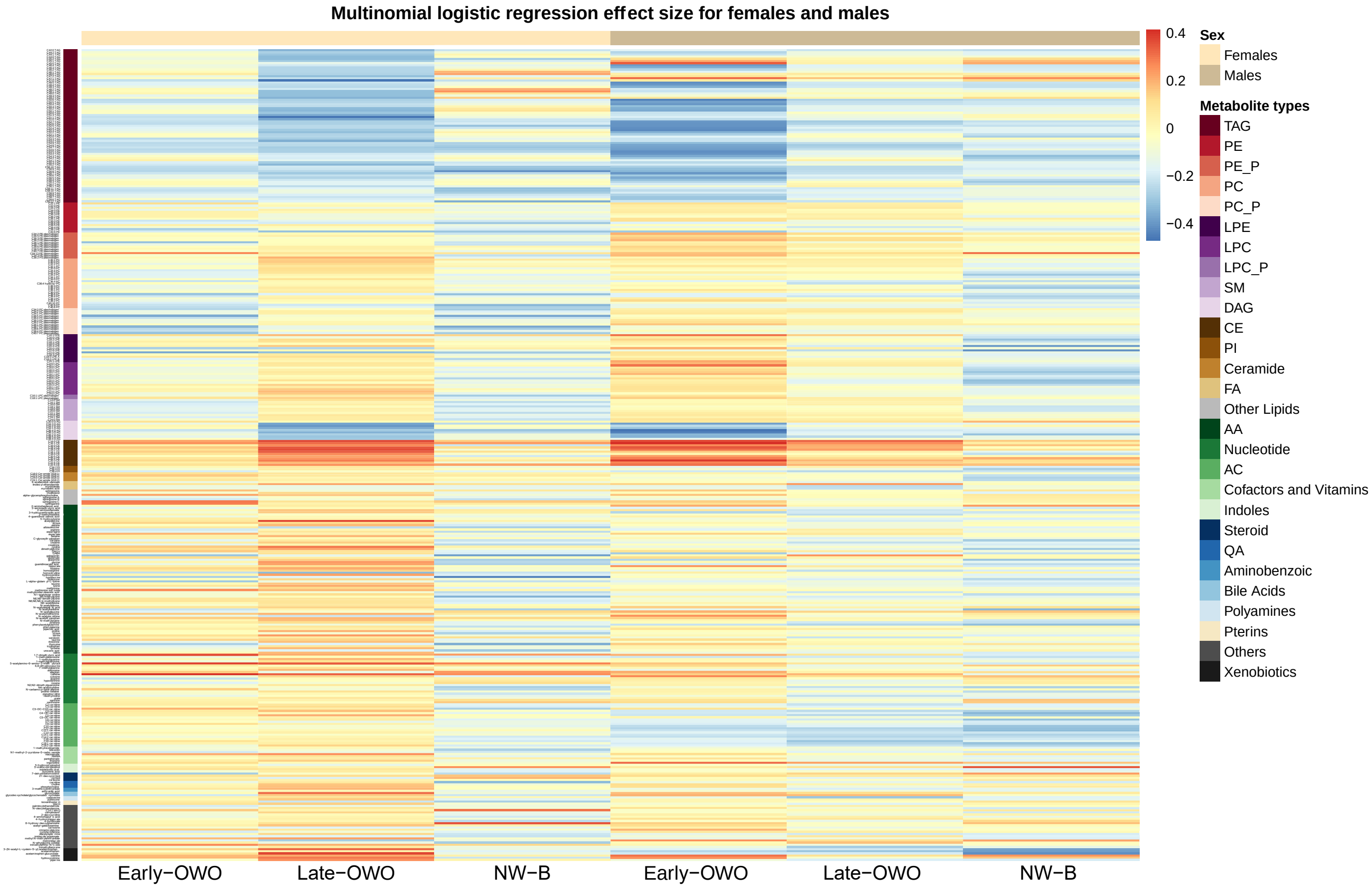

Supplementary Figure S2. Longitudinal trajectory analysis - sensitivity analysis: Heatmap of the effect size of metabolites on BMI trajectory (4 groups) for females (first 3 columns) and males (last 3 columns) respectively on the horizontal axis. The vertical axis represents the 376 metabolites which are ordered based on metabolite types. The effect size of the association between each metabolite and child BMI trajectory is the beta coefficient from the multinormal logistic regression (reference group is NW-A, i.e. the purple line in Figure 1B) with the sex*metabolite interaction term. The color scheme for effect size is continuous such that red and blue indicates positive and negative association respectively. The intensity of the color represents the magnitude of the association.

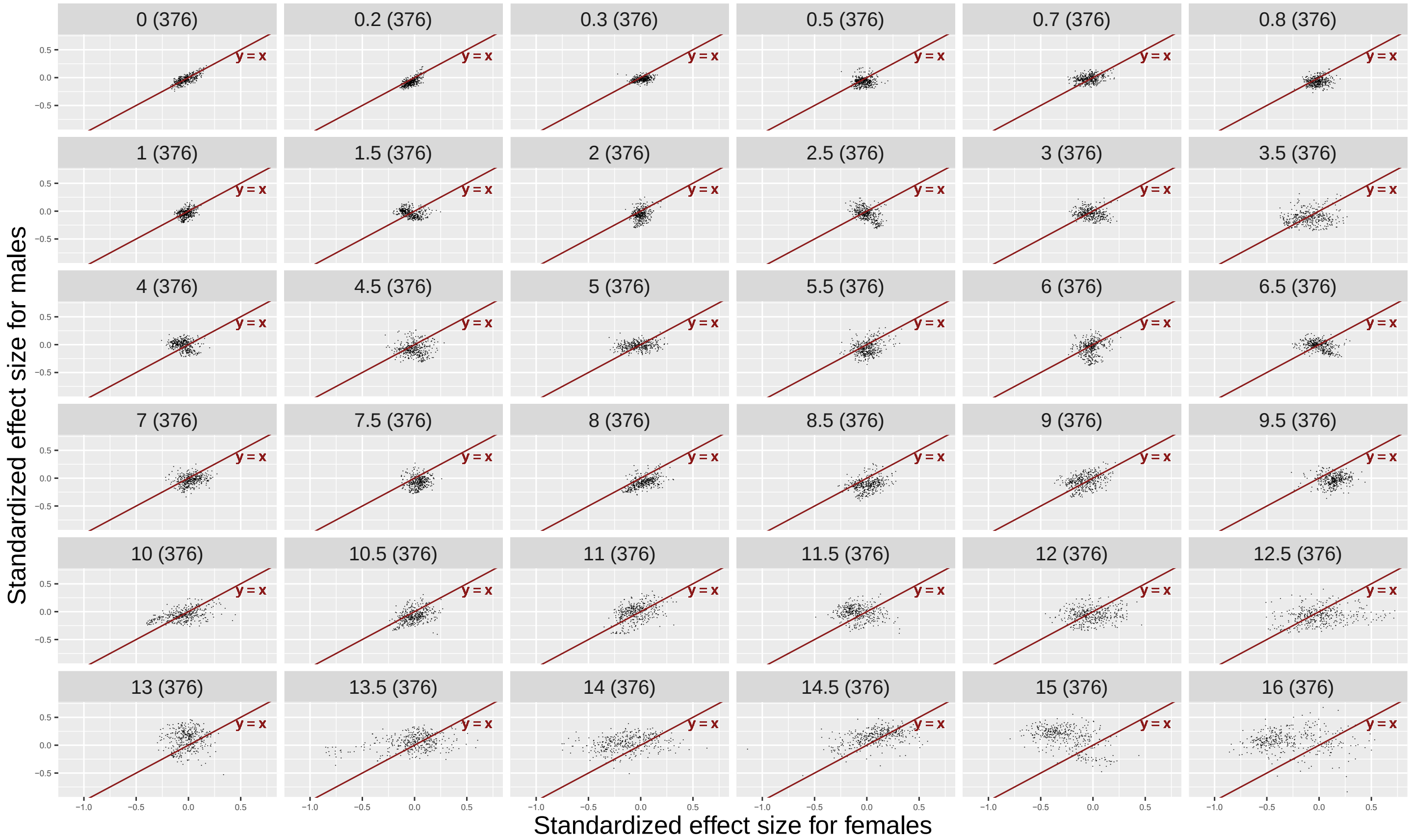

Supplementary Figure S3. Time-window specific analysis: Scatter plot of the standardized effect size in males vs. females for BMI. The y=x lines are shown as reference in slanted dark red lines.

A.

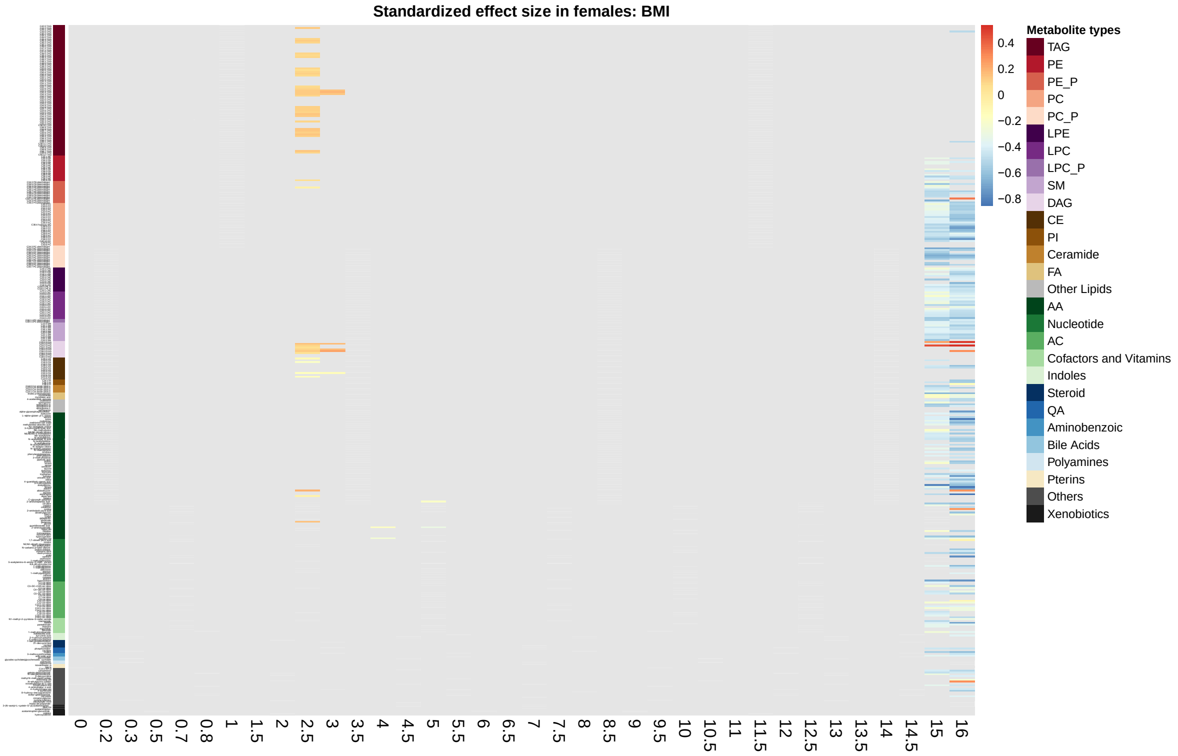

B.

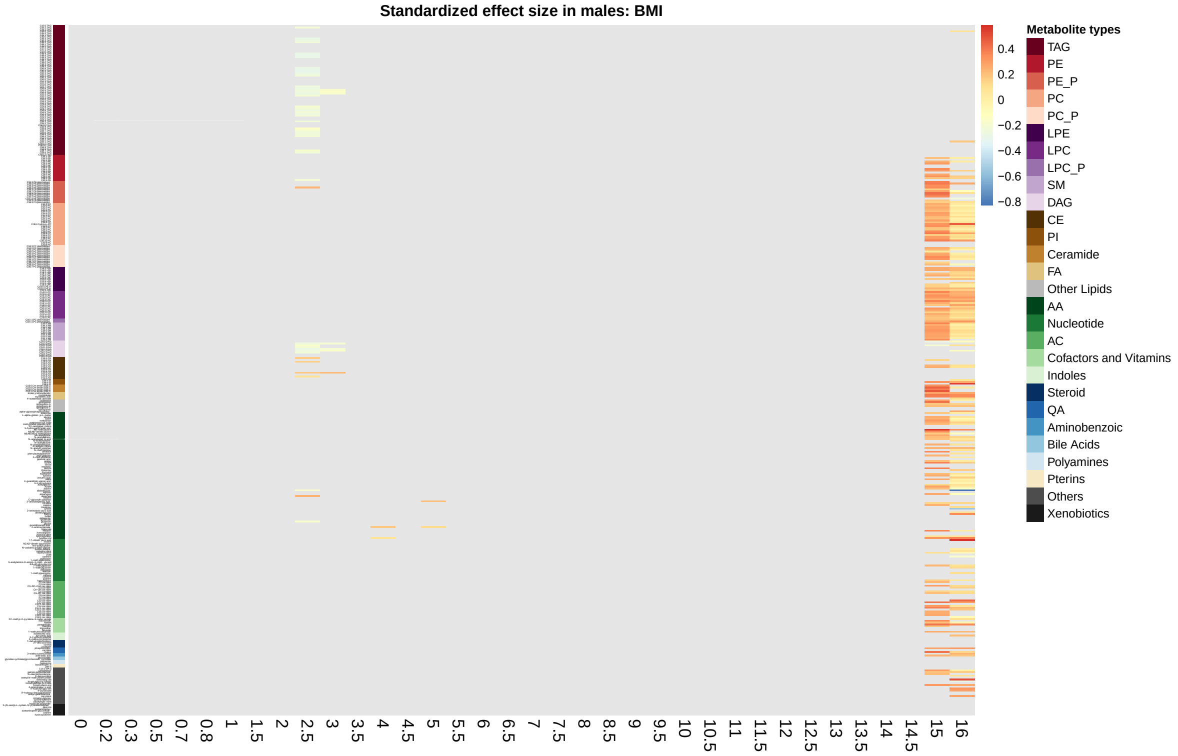

Supplementary Figure S4. Time-window specific analysis: Heatmap of cord metabolites' effect size for females (Panel A) and males (Panel B) on BMI masked by non-significance of interaction term FDR. The vertical axis represents the 376 metabolites which are ordered based on metabolite types. Effect sizes are standardized by dividing the beta coefficient by the standard deviation of BMI for that sex at that time-window. The color scheme is continuous such that red and blue indicates positive and negative association respectively. The intensity of the color represents the magnitude of the association. The grey color indicates where there is no statistically significant difference between females' and males' effects of that metabolite at that age range (metabolite-by-sex interaction term FDR > 0.05).

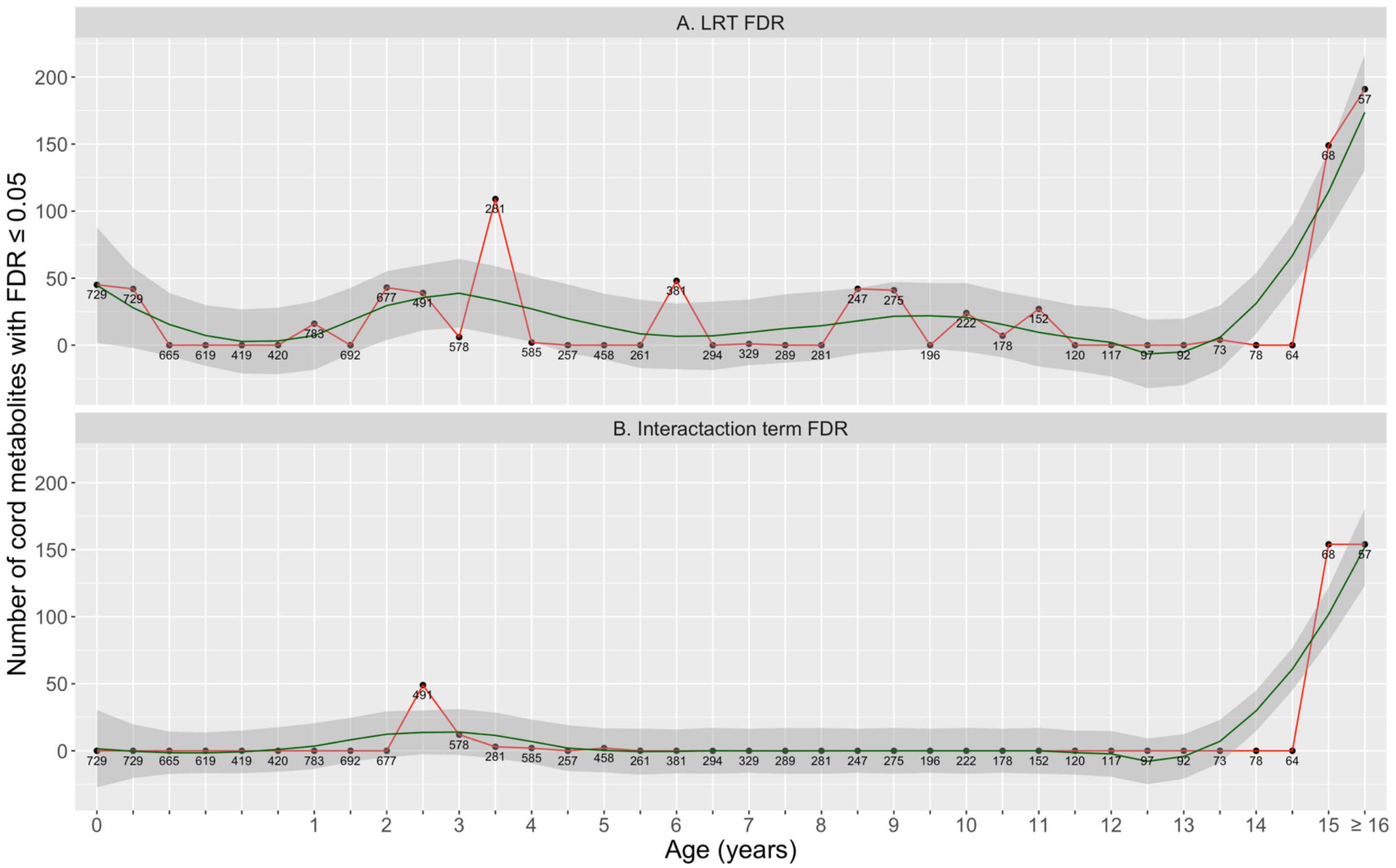

Supplementary Figure S5. Time-window specific analysis – sensitivity analysis further adjusted for cesarean section: Number of significant metabolites at each time-window based on LRT FDR (Panel A), number of metabolites that have significantly different effects between two sexes at each time-window based on metabolite-by-sex interaction term FDR (Panel B). Annotated numbers are the sample size (number of subjects) available at each time-window. Green lines are the LOWESS smoothing curves. Potential critical growth ages (peaks in Panel A) are around age 0 (birth), 1-3.5, 6, 8-11 and ≥15.

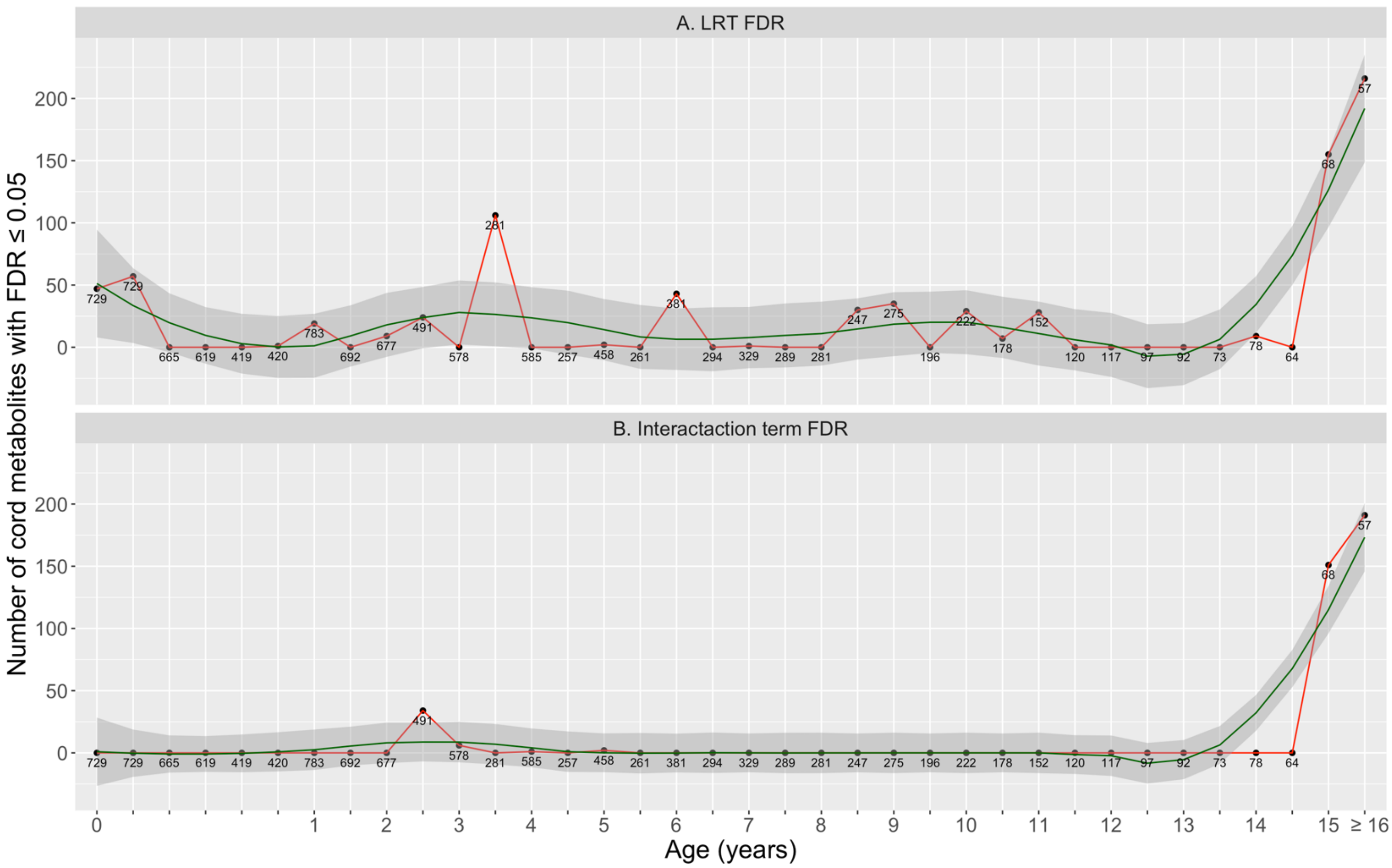

Supplementary Figure S6. Time-window specific analysis – sensitivity analysis further adjusted for breastfeeding: Number of significant metabolites at each time-window based on LRT FDR (Panel A), number of metabolites that have significantly different effects between two sexes at each time-window based on metabolite-by-sex interaction term FDR (Panel B). Annotated numbers are the sample size (number of subjects) available at each time-window. Green lines are the LOWESS smoothing curves. Potential critical growth ages (peaks in Panel A) are around age 0 (birth), 1-3.5, 6, 8-11 and ≥15.

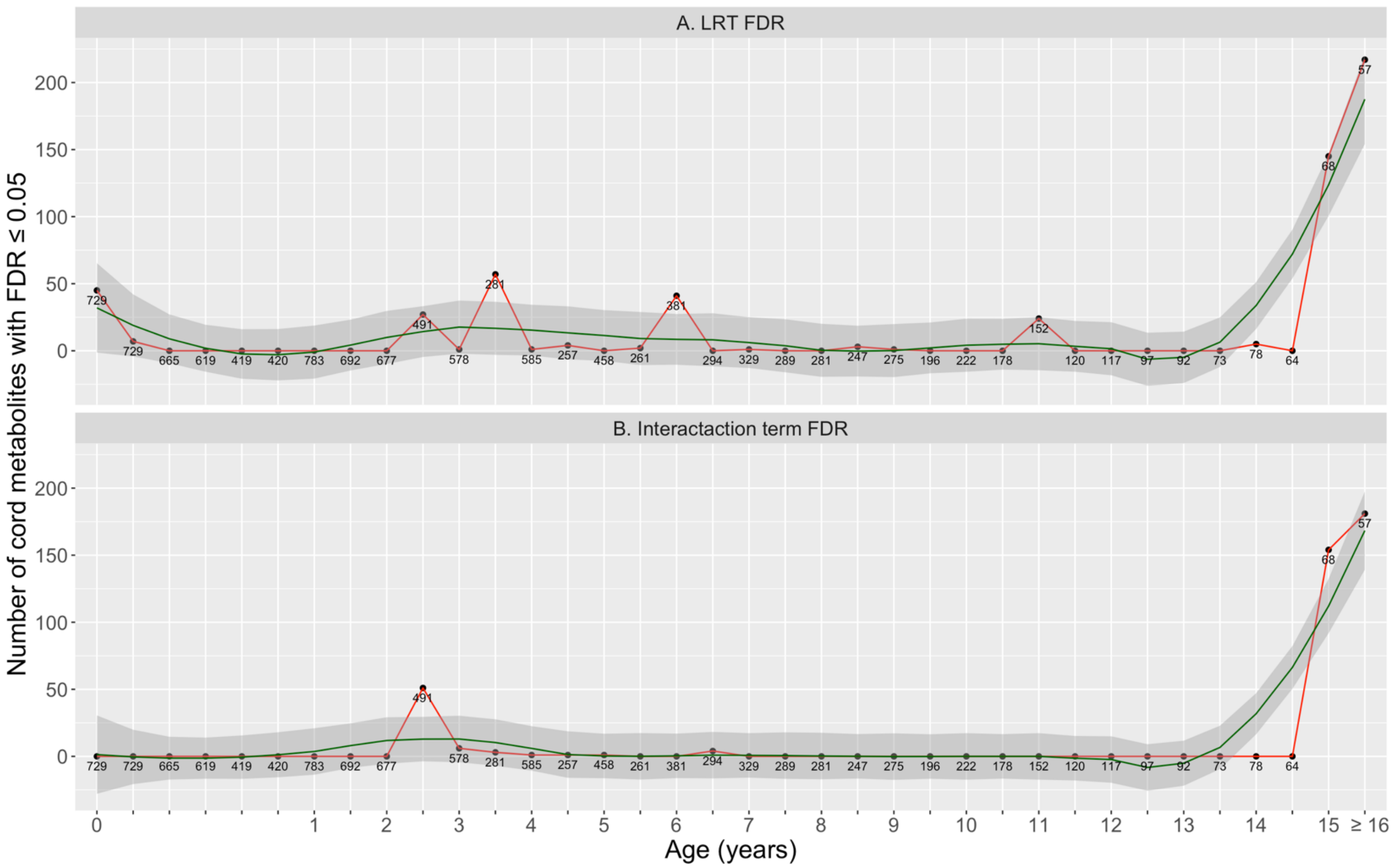

Supplementary Figure S7. Time-window specific analysis – sensitivity analysis further adjusted for birthweight: Number of significant metabolites at each time-window based on LRT FDR (Panel A), number of metabolites that have significantly different effects between two sexes at each time-window based on metabolite-by-sex interaction term FDR (Panel B). Annotated numbers are the sample size (number of subjects) available at each time-window. Green lines are the LOWESS smoothing curves. Potential critical growth ages (peaks in Panel A) are around age 0 (birth), 2-4, 6, 11 and ≥15.

A.

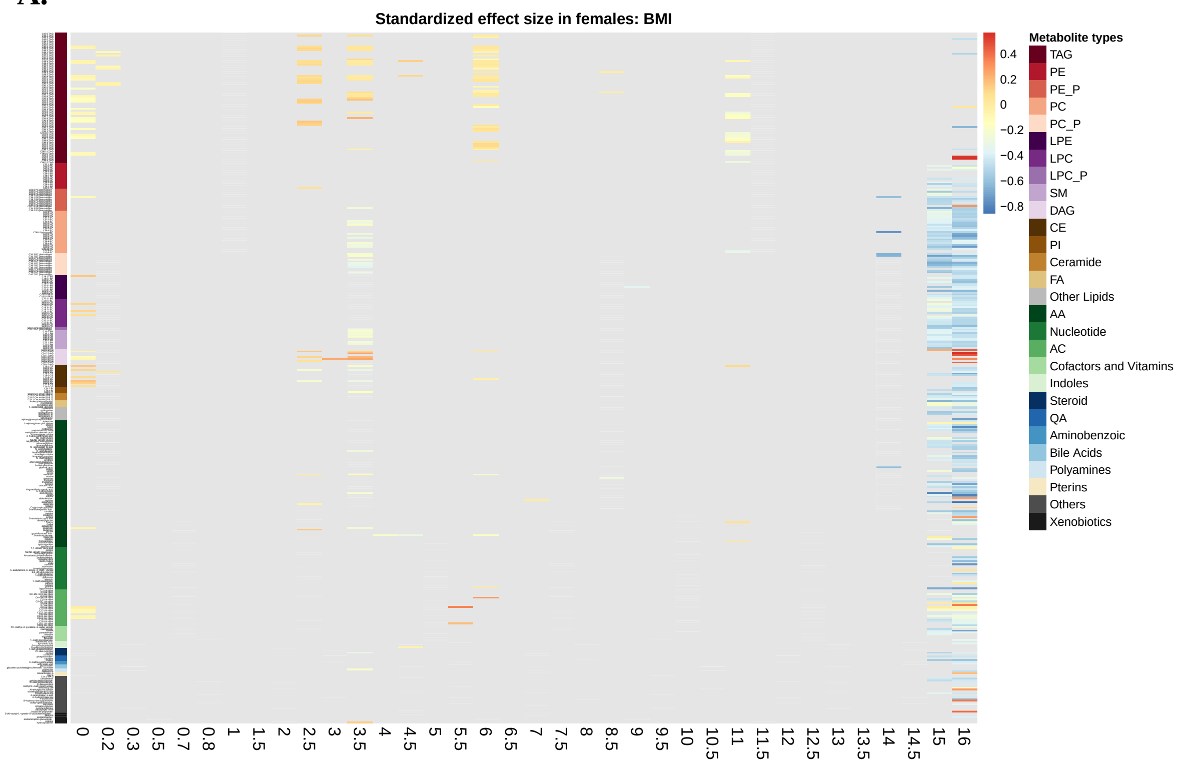

B.

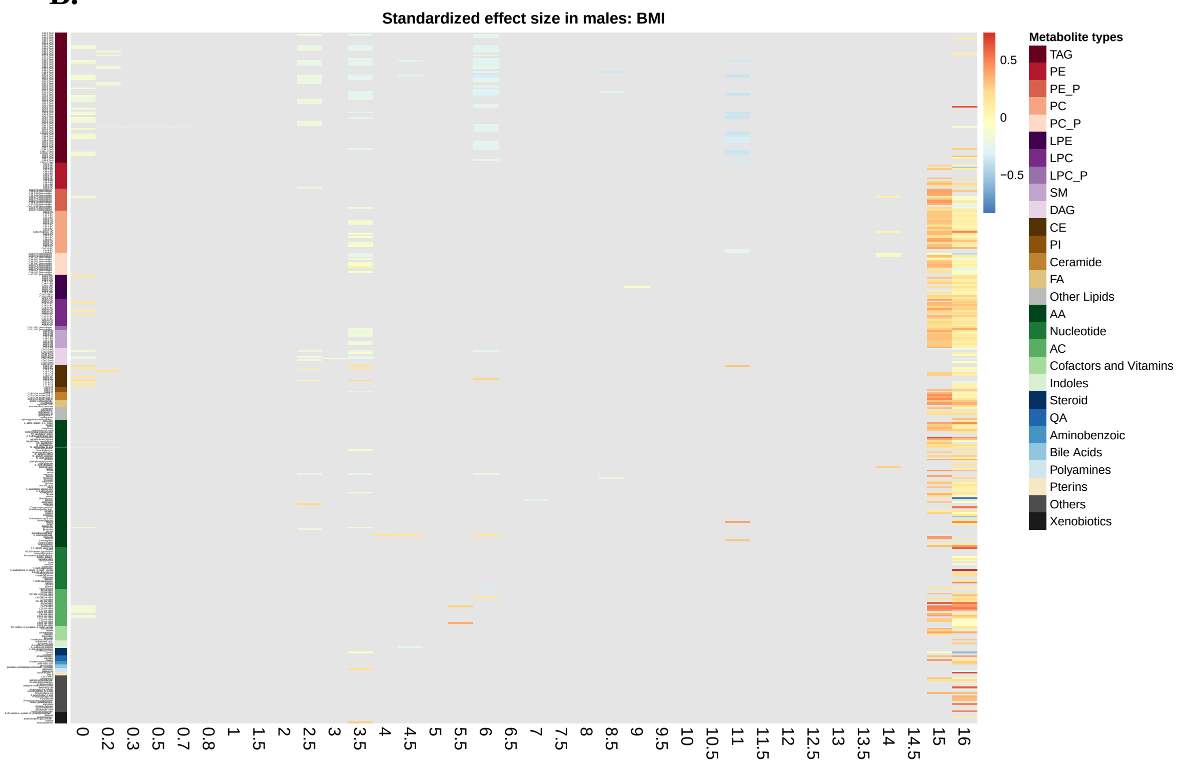

Supplementary Figure S8. Time-window specific analysis – sensitivity analysis further adjusted for birthweight: Heatmap of cord metabolites' effect size for females (Panel A) and males (Panel B) on BMI masked by non-significance. The vertical axis represents the 376 metabolites which are ordered based on metabolite types. Effect sizes are standardized by dividing the beta coefficient by the standard deviation of BMI for that sex at that time-window. The color scheme is continuous such that red and blue indicates positive and negative association respectively. The intensity of the color represents the magnitude of the association. The grey color indicates where the overall effect of that metabolite at that time-window is not statistically significant (LRT FDR > 0.05).
